# Biomedical Prevention: State of the Science

**DOI:** 10.1093/cid/ciu297

**Published:** 2014-07-01

**Authors:** Sheena M. McCormack, Mitzy Gafos, Monica Desai, Myron S. Cohen

**Affiliations:** 1 Medical Research Council, Clinical Trials Unit at University College London, United Kingdom; 2 Department of Epidemiology, University of North Carolina at Chapel Hill

**Keywords:** preexposure prophylaxis, postexposure prophylaxis, treatment as prevention, PrEP, TasP

## Abstract

Preexposure prophylaxis (PrEP) and treatment as prevention (TasP) involve the use of antiretroviral (ARV) drugs by human immunodeficiency virus (HIV)-negative and -positive individuals to reduce HIV acquisition and transmission, respectively. Clinical science has delivered a consistently high effect size for TasP and a range from 0%–73% reduction in incidence across placebo-controlled PrEP trials. However, the quality of evidence for PrEP compares favorably with evidence for postexposure prophylaxis (PEP). It is clear from treatment programs and PrEP trials that daily adherence presents challenges to a large proportion of the population. Although there are factors associated with inconsistent use (ie, younger age), they do not assist clinicians at the point of care. There are additional provider concerns about PrEP (covering cost of drug and delivery, undermining condom promotion, and facilitating resistant strains) that have delayed widespread acceptance. These issues need to be addressed in order to realize the full public health potential of antiretrovirals.

The first demonstration that human immunodeficiency virus (HIV) could be prevented with a biomedical intervention was the dramatic reduction of transmission from mother to child in 1994 with use of a single drug, zidovudine, in a complex regimen that was not practical to implement in all settings [1]. Randomized placebo-controlled trials of more practical regimens were undertaken, eliciting controversy in the literature but providing the necessary evidence for the World Health Organization (WHO) to modify guidelines and incorporate a range of options. More potent regimens have followed; however, in 2012 an estimated 260 000 babies in low- and middle-income countries were infected with HIV from their mother [2].

Scientific endeavors have delivered robust evidence for the biological efficacy of preexposure prophylaxis (PrEP) [3] and treatment as prevention (TasP) [4]. There is cautious optimism for tenofovir 1% vaginal microbicide [5] and a glimmer of hope for combination vaccine regimens, with significant protection demonstrated in 1 trial (RV144) [6]. In contrast, the evidence base for postexposure prophylaxis (PEP) following sexual exposure is weak, with supporting preclinical and cohort data only (mainly occupational) [7].

This review, which is based on the 2013 International Association of Providers of AIDS Care (IAPAC) summit “Controlling the HIV Epidemic With Antiretrovirals,” held in London, focuses exclusively on the use of antiretroviral (ARV) drugs to prevent onward transmission to sexual partners (TasP), as postexposure prophylaxis after sex (PEPSE), and before the event (PrEP, microbicides). We identify the challenges in translating the science for prescribers and users, and the difficulty that the lack of precision around the estimates of effect presents for progressing drugs that are currently in early development.

## EVIDENCE FOR EFFICACY

Academic groups working independently of each other have collected the evidence for each biomedical intervention. Whether by serendipity or Gilead Sciences' foresight in making tenofovir and Truvada accessible, there is a comprehensive portfolio for these ARV drugs when used as oral and topical PrEP. This includes preclinical challenge experiments [8, 9], pharmacokinetic data with and without a pharmacodynamic endpoint [10–14], and randomized placebo-controlled clinical trial data in a range of diverse populations [5, 15–20].

## THE CHALLENGE OF NO TRIAL: POSTEXPOSURE PROPHYLAXIS

The evidence base for PEPSE is at the bottom of the Grading of Recommendations Assessment, Development, and Evaluation (GRADE) system for rating evidence [21], relying heavily on preclinical data and cohort data (mainly following occupational injury) [7]. Despite this, it is widely available in all countries using ARV drugs licensed for the treatment of HIV. The 72-hour limit for starting PEPSE after exposure is derived from preclinical data [22, 23] but endorsed by clinical data collected after known exposure [24–26]. Earlier is clearly better. Therefore, it is not appropriate to assess time to start in a randomized trial; however, surely it is time to assess shorter regimens given the evidence that HIV is disseminated 5–10 days after exposure [27].

## THE ADVANTAGE OF THE SINGLE TRIAL AND A SURROGATE MARKER

When HIV Prevention Trials Network (HPTN) 052 randomized the first participant to early or deferred antiretroviral therapy (ART), there was already a body of evidence that supported negligible risk of onward transmission from positive individuals with undetectable virus in the plasma [28, 29]. The evidence available in 2008 was sufficient to lead the Swiss to issue a statement to this effect [30]; this statement led to considerable controversy when presented at the XVII International AIDS Society Conference in Mexico City [31]. The controversy created uncertainty, and the majority of healthcare workers felt uncomfortable advising patients they were not infectious and deemed it necessary to mention the caveats to them (eg, unable to extrapolate from cohort populations due to differing sexual practices, virus in the genital compartment if not the plasma, the possibility that other sexually transmitted infections [STIs] are present to increase susceptibility or viral shedding). HPTN 052 changed that by providing a single, very high estimate of effect, that is, 96%, together with the explanation that the single infection that was transmitted most likely occurred before the positive individual became undetectable [28]. The caveats have not entirely disappeared, but the message for the individual considering this option is clear, with the WHO strongly recommending initiation of treatment for the HIV-infected partner in a discordant sexual relationship, regardless of CD4 count or WHO stage of disease, so as to prevent onward transmission [32]. This recommendation is for serodiscordant couples of any orientation, although data for men who have sex with men (MSM) are lacking [33].

The public health benefit of TasP is not so clear and is being specifically addressed in 4 randomized trials being conducted in heterosexual populations in countries in sub-Saharan Africa with generalized epidemics [34, 35]. However, the incidence of HIV in gay and other MSM is rising in several countries that have robustly implemented ART programs, leading some experts to conclude that TasP alone will not eradicate HIV in these focused epidemics [33, 36, 37].

## THE PROS AND CONS OF MULTIPLE, WIDE-RANGING ESTIMATES FOR PrEP

Statistically significant reduction in HIV incidence is a clear endpoint, and the demonstration of this in diverse populations leaves no room for doubt that daily oral Truvada has biological efficacy. However, there is uncertainty about the size of the effect, as the range of benefits observed in clinical trials goes from no benefit to 73% reduction in HIV incidence compared with placebo. This uncertainty makes it difficult to articulate a simple, clear message for prescribers and users of PrEP regarding how much protection daily Truvada will provide, although it is clear that it can only work if it is taken. Even though the differences between the trials can be explained by the differences in the proportion who were taking the drug [38], there are distracting caveats in the literature that include lower levels of drug in the female genital tract [39], the vulnerability of rectal epithelial cells [40, 41], and the presence of facilitating STIs [42].

The uncertainty around the effect size for Truvada, the only licensed drug for PrEP, also presents a challenge for those wishing to demonstrate the effectiveness of alternative drugs for PrEP. Where biological efficacy is uncertain, the most robust control remains a placebo [43]. This is why the tenofovir vaginal microbicide trial FACTS001, the “before and after sex” Truvada PrEP trial iPerGay, and the 2 dapivirine vaginal ring studies (ASPIRE and the Ring) are using placebo controls [44]. In the absence of a validated surrogate marker for efficacy, it is difficult to see a future for oral PrEP that does not include large noninferiority trials with extensive pharmacokinetic sampling in order to determine the proportion taking the drug in each group so that the analyses can be adjusted for differences in adherence. What level of efficacy should these designs assume for the daily Truvada control? Should it be 90%, as suggested by the 2 nested case-control studies in iPrEx and Partners? Or somewhere between the 44% seen in iPrEx and the 73% seen in Partners? The choice is likely to be driven by practicalities such as the sample size that can be achieved, which can be a recipe for underpowered trials. Interpretation of the results of such trials is guaranteed to be challenging [45].

## ADHERENCE BEHAVIOUR

TasP, PrEP, microbicides, and PEPSE are better understood as biobehavioral interventions as they are all user dependent, whether they are pills that need to be taken at a certain frequency, microbicide gels or rings that need to be inserted and left in situ for specified periods, or injectables that require clinic attendance at particular intervals.

Clinical trials of PrEP and ARV-based microbicides have demonstrated that although adherence to visits was high in all trials, adherence to product measured objectively through detection of drug varied considerably [16–18, 20, 46]. In roll-out programs, uptake of ARV prevention options is more likely to be driven by need than by the benefits inherent in clinical trial participation [47, 48]. Later in this supplement, Amico and Stirratt describe the evidence highlighting demographic, psychological, and socioecological factors related to product use in placebo-controlled trials to date [49]. Although evidence from FemPrEP and VOICE suggest insufficient levels of adherence to daily study products, the underlying reasons for nonadherence are not clear. Until this is understood, we should not dismiss the potential of daily oral PrEP in similar populations. Amico and Stirratt highlight the substantial gaps that remain in our understanding of how and why open-label PrEP will be adhered to, interrupted, and discontinued. These gaps hinder our ability to define clear-cut messages for users in open-label studies and in real life. Although adherence to an ARV prevention option will be less obscured by unknown safety and efficacy of a study product, how will healthcare providers and users interpret the level of efficacy? And what impact will the uncertainty around the effect size have on policy, practice, and adherence?

And what of adherence to TasP in the absence of real-time feedback of viral load measurements to identify those who need additional support and to reinforce the value of adherence? The additional benefit of negligible risk of onward transmission may add incentive to receive ART, although, interestingly, 13% of the HPTN 052 cohort chose to remain off ART 18 months after unblinding [50].

In addition to noting the extent to which product use in trials may differ from product adherence in real life, it is likely that usage patterns will be influenced by product modality and regimen. CAPRISA 004 evaluated a “before-and-after” dosing of topical PrEP, whereas FemPrEP and VOICE evaluated daily dosing strategies of oral and oral–topical PrEP, respectively. Prior efficacy trials assessed a simple “before-sex” application of non-ARV vaginal microbicide gel. It is important not to dismiss the large body of evidence on product use from these trials because of a lack of biological measures of adherence. Several non-ARV microbicide trials used multiple measurement methods [51] and reported similar disparities between self-reported point prevalent product use and composite or indirect measures of product use, as seen in ARV-based trials between self-reported point prevalent use and drug level counts. In spite of the limitations of the clinical trial setting, it was possible to collect data that went beyond acceptability to provide a more holistic picture of how women incorporated gel into their lives and partnerships across cultures that will be relevant to real-life use [52].

## ACTUAL RISK AND PERCEIVED RISK

We already know from models of HIV transmission, repeated cross-sectional studies, and surveillance data collected in the United Kingdom and the United States that risk behaviors and markers of risk behavior, such as STIs, continue to increase among MSM [53–58]. This resurgence in STIs compared with the early years of HIV infection predates the introduction of PrEP. New diagnoses of HIV are also increasing in key populations with stable epidemics. Although use of new diagnoses to estimate incidence is subject to ascertainment bias due to increased testing, the incidence of HIV in the placebo group of several HIV prevention trials has been higher than anticipated [5, 20]. Therefore, it seems likely that the increase in reported condomless sex is real and prevalent among HIV-positive and -negative populations. The drivers are complex and include a change in the sexual environment, with networks broadening through social networking sites, changes in sexual risk behaviors [59, 60], and the impact of modifying a simple “no-sex or condoms-only” instruction to more nuanced “safer-sex” messages. The role that repeatedly testing HIV negative while having condomless sex plays in reinforcing an individual's perception of their low-risk status merits evaluation.

Science has facilitated a precise measure of risk of transmission following sexual exposure based on a composite of the chance that the person was HIV positive and on the type of sex. For example, in central London, where prevalence in MSM is 8.1%, the risk from unprotected receptive anal intercourse is calculated to be 1 in 1112 with a partner of unknown status [61]. This creates an impression of low risk, even more so for insertive anal intercourse where the calculated risk is 1 in 20 408. Are these messages appropriate when we see mean viral loads of 8 million copies/mL in seroconvertors [62] who believe themselves to be HIV negative based on their most recent test?

The longitudinal self-reported risk behavior observed in the iPrEx cohort [63] suggests that the need for PrEP is more likely to be periodic than constant. The extent to which PrEP increases condomless sex with and without an increase in the number of partners has yet to be characterized. Although PrEP will render most, if not all, sex acts protected against HIV, it will not prevent other STIs and may not prevent HIV transmission when the viral load is very high, as is the case in seroconversion. The impact of “risk compensation” on effectiveness and cost-effectiveness cannot be measured in placebo-controlled trials when participants know they could be on an inactive drug. Limited data from open-label extensions of the placebo-controlled studies in heterosexuals are reassuring [64], but this may not extrapolate to populations with a greater frequency of partner change, as is the case with some MSM populations [65].

## COST SAVINGS, COST EFFECTIVENESS, COST BENEFIT

Wilson and Fraser provide a detailed review of the economics of ARV drugs for prevention in this supplement [66]. They have highlighted the 4-fold difference between current spending on AIDS care and treatment and the funds required to treat everyone living with HIV [37]. There are opportunities for efficiencies (task shifting, declining drug costs, cheaper diagnostics), and it is encouraging to see several low- and middle-income countries that are now independent of external assistance and able to consider the cost effectiveness of these new strategies, with projected cost savings for HIV treatment programs in the long term. Nonetheless, there remain high-burden countries that cannot contemplate new options regardless of how good they appear to be, as they have not yet managed to treat all those with CD4 <200 cells/mm^3^. In all settings, the cost benefit of each strategy has to be considered against the alternatives in the context of finite resources within the healthcare sector.

## CONCLUSION

The state of the science for biomedical interventions ranges from weak (for PEPSE), to robust with complex messages (for PrEP), to robust and clear (for TasP). Postexposure prophylaxis for sexual exposure has been available for some time, and it was possible to advocate for this with virtually no evidence. For TasP, the US President's Emergency Plan for AIDS Relief, WHO, International Association of Providers of AIDS Care, International AIDS Society, US Agency for International Development, and US Department of Health and Human Services have embraced a much more aggressive promotion of ART, in part, for the public health potential. The policy and practice response to PrEP, in contrast, has been slow and likely reflects the more complex messages that accompany this biomedical intervention, which has proven to be more behavioral than anticipated. Guidelines and position statements have been developed; however, uptake, as measured by prescriptions in the United States, has been poor. Now is not the time to stand still in admiration of what has been achieved but rather to double our efforts to answer the questions that will accelerate implementation of the most appropriate strategies for each epidemic setting in order to achieve a 50% reduction in HIV incidence. Failure is not an option.
